# Addiction-related brain networks identification via Graph Diffusion Reconstruction Network

**DOI:** 10.1186/s40708-023-00216-5

**Published:** 2024-01-08

**Authors:** Changhong Jing, Hongzhi Kuai, Hiroki Matsumoto, Tomoharu Yamaguchi, Iman Yi Liao, Shuqiang Wang

**Affiliations:** 1 Shenzhen Institutes of Advanced Technology, Chinese Academy of Sciences, Shenzhen, China; 2 Faculty of Engineering, Maebashi Institute of Technology, Maebashi, 371-0816 Japan; 3 Gunma University of Health and Welfare, Maebashi, Japan; 4 University of Nottingham Malaysia Campus, Semenyih, Malaysia

**Keywords:** Brain connectivity, Graph diffusion, Nicotine addiction, Generative learning

## Abstract

Functional magnetic resonance imaging (fMRI) provides insights into complex patterns of brain functional changes, making it a valuable tool for exploring addiction-related brain connectivity. However, effectively extracting addiction-related brain connectivity from fMRI data remains challenging due to the intricate and non-linear nature of brain connections. Therefore, this paper proposed the Graph Diffusion Reconstruction Network (GDRN), a novel framework designed to capture addiction-related brain connectivity from fMRI data acquired from addicted rats. The proposed GDRN incorporates a diffusion reconstruction module that effectively maintains the unity of data distribution by reconstructing the training samples, thereby enhancing the model’s ability to reconstruct nicotine addiction-related brain networks. Experimental evaluations conducted on a nicotine addiction rat dataset demonstrate that the proposed GDRN effectively explores nicotine addiction-related brain connectivity. The findings suggest that the GDRN holds promise for uncovering and understanding the complex neural mechanisms underlying addiction using fMRI data.

## Introduction

Addiction is a disease characterized by seeking compulsive drugs. Smoking addiction is not only the most common drug addiction behavior in humans worldwide [1, 2], but also considered to be one of the leading causes of death and disease in the world [3]. Clinical studies have shown that long-term exposure to nicotine can lead to changes in brain structure and function [4]. However, few studies have focused on the changes in global brain functional networks caused by long-term exposure to nicotine, which are associated with severe damage to brain circuits [5], especially in acute nicotine withdrawal [6]. To better understand smoking behavior and help improve the treatment of nicotine addiction, key functional connectivity and mechanisms of addiction that are altered by acute nicotine withdrawal and recovery need to be identified.

Imaging studies have revealed neurochemical and functional changes in the brains of addicted individuals, providing new insights into the mechanisms of addiction. Functional magnetic resonance image (fMRI) is currently our most powerful tool [7] for non-invasive functional imaging of the whole brain [8]. The development of magnetic resonance imaging has transformed the study of neuroanatomy, enabling for the first time well-contrasted in vivo experiments in different brain regions. The brain network is divided into different brain regions by anatomical structure and connected together, and the functional brain network shows its complex neuron communication and signal transmission mode. Thanks to the advancement of modern imaging technology and advanced medical image analysis methods [9], the pattern of this complex neural signal can be analyzed from functional images, which reveals neuronal activities related to behavior and cognition, as well as brain diseases.

In brain imaging computing, artificial intelligence technology [10, 11] based on machine learning [12] can effectively improve the efficiency [13, 14] of doctors’ treatment and the accuracy of diagnosis. Convolutional neural networks reduce the dimensionality of medical image data through convolution operators, which can effectively identify patterns in neuroimaging. Generative adversarial strategies [15–18] can simulate the real distribution of data [19], reduce the interference caused by noise, and enhance the robustness of the model. Generative artificial intelligence [20–22] can be applied to brain network analysis [23, 24] to help better understand the function and structure of the brain [25]. In brain network analysis, generative artificial intelligence can be used to generate simulated data to explore different types of neurons and connections between neurons, which can help to better understand brain networks [26]. It can also be used to simulate the transmission of signals between neurons to help better understand the interaction and information transmission between neurons [27]. Generative artificial intelligence can be used in neuroimaging to help better interpret neuroimaging data and provide information about the structure and function of brain networks [28]. Generative artificial intelligence can be used to denoise and de-artifact fMRI data, thereby improving the accuracy and reliability of brain network analysis [29]. Generative artificial intelligence has broad application prospects in brain network analysis, which can help to better understand the function and structure of the brain, and provide new opportunities and methods for neuroscience research [30].

Related work: The strategy of generative adversarial learning [31, 32] can be easily applied to the field of brain imaging [33]. Conte et al. [34] developed a generative adversarial networks (GAN) based on the pix2pix framework for a brain tumor segmentation model. Pan et al. [35] designed a disease image-specific network framework (DSNet) to model the specificity of disease images with spatial cosine implicits. Bo et al. [36] developed a multi-tracer positron emission tomography (PET) synthesis model for the task of generating multi-tracer PET volumes from single-tracer PET. Jiao et al. [37] study the cross-modal generation task of MRI and propose an end-to-end self-supervised GAN model for MRI synthesis.

With the rapid development of diffusion models in the field of cross-modal generation, such as denoising diffusion probabilistic model (DDPM) and denoising diffusion implicit model (DDIM), more and more studies have applied diffusion models to the field of brain imaging research. Wolleb [38] adopted a DDIM-based anomaly detection model to achieve anomaly detection tasks for brain tumor images. Pinaya [39] used DDPM/DDIM to realize the detection and segmentation of diseases such as brain tumors and cerebral hemorrhage, and performed better than Transformer on synthetic data and real data. DDPM/DDIM shows better performance on disease tasks such as brain tumor and cerebral hemorrhage. Khader [40] proposed a diffusion model Medical Diffusion applied to 3D images for 3D brain image generation. Chung et al. [41] used score-based accelerated MRI reconstruction to produce highly accurate results on the MRI reconstruction task. However, the existing methods are still difficult to effectively obtain the brain connections related to nicotine addiction from fMRI images.

To address the above issues, this paper proposed a graph diffusion reconstruction network (GDRN) to capture brain connections associated with nicotine addiction from fMRI data of addicted rats. The diffusion reconstruction module effectively maintains the unity of the data distribution in the latent space by reconstructing the training samples. This module enhances the reconstruction of nicotine addiction-related brain networks, allowing the model to learn more subtle distribution differences. This allows the model to effectively capture addiction-related brain connections.

## Method

**Fig. 1:**
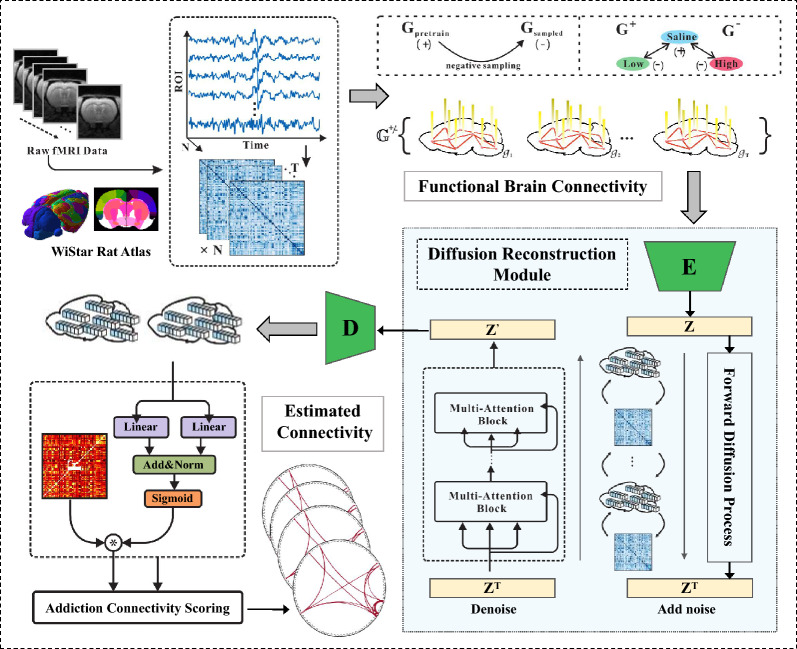
The structure of the proposed Graph Diffusion Reconstruction Network

In order to generate more effective addiction-related brain networks, so as to capture the characteristics of addiction and finally detect addiction-related brain connections, a reconstruction network with graph diffusion is proposed for the generation of addiction-related brain connections. The overall architecture of the framework is shown in Fig. 1, which includes functional brain network construction, brain network diffusion reconstruction module and addiction-related brain connection detector. The following mainly introduces the proposed brain network diffusion reconstruction module.

From a probabilistic modeling point of view, the key defining characteristic of a generative model is that it is trained in such a way that its samples ${\tilde{x}} \sim p_\theta ({\tilde{x}})$ come from the same distribution $x\sim p_\theta (x)$ as the training data distribution. Energy-based models do this by defining an unnormalized probability density over the state space. However, if these methods perform Markov Chain Monte Carlo (MCMC) sampling during both training and inference, a slow iterative process is required. The Denoising Diffusion Probabilistic Models (DDPMs) define a forward diffusion phase. In this method, the input data are gradually perturbed in several steps by adding Gaussian noise, and then learns the backdiffusion process. Data are recovered by reversing this noise process.

Diffusion models have strong pattern coverage and quality of generated samples. This brain network diffusion reconstruction module combines and applies this technique to the generation of addiction-related brain networks. The ability to effectively extract latent variables with the help of diffusion models captures addiction-related representations for the detection of addiction-related brain connections in the next step.

Inspired by DDPM, this module also adopts the diffusion forward process and reverse diffusion process when reconstructing addiction-related brain networks. The model was used to generate the addiction brain network of rats in the normal saline group, the low-concentration nicotine group, and the high-concentration nicotine group.

In the forward process, given the initial observation value $x_0 \sim q\left( x_0\right)$, it is defined as a Markov chain and a diffusion process is performed. And update the conditional probability of the current sample $x_t$ at each time step. Finally, from the conditional probabilities at all time steps, the probability density function for $x_0$ can be calculated. Taking $\alpha _t=1-\beta _t$ and ${\bar{\alpha }}_t=\prod _{s=0}^t \alpha _s$ as the premise, in order to be able to sample any step of the noise latency under the input $x_0$ condition, the forward formula can be expressed as follows:

1 $$\begin{aligned}{} & {} q\left( {\textbf{x}}_t \mid {\textbf{x}}_0\right) =N\left( {\textbf{x}}_t; \sqrt{{\bar{\alpha }}_t} {\textbf{x}}_0,\left( 1-{\bar{\alpha }}_t\right) {\textbf{I}}\right) , \end{aligned}$$

2 $$\begin{aligned}{} & {} {\textbf{x}}_t=\sqrt{{\bar{\alpha }}_t} {\textbf{x}}_0+\sqrt{1-\overline{\alpha _l}} \epsilon , \end{aligned}$$

where ${\textbf{I}}$ is the identity matrix ${\mathcal {N}}(x;\mu ,\sigma )$ representing the normal distribution with mean $\mu$ and covariance $\sigma$.

In the backward pass, the forward pass is reversed to get a sample from $q(x_0)$. For this purpose, it has the following formula:

3 $$\begin{aligned}{} & {} p_\theta \left( {\textbf{x}}_{0: T}\right) =p\left( {\textbf{x}}_T\right) \prod _{t=1} p_\theta \left( {\textbf{x}}_{t-1} \mid {\textbf{x}}_t\right) , \end{aligned}$$

4 $$\begin{aligned}{} & {} p_\theta \left( {\textbf{x}}_{t-1} \mid {\textbf{x}}_t\right) ={\mathcal {N}}\left( {\textbf{x}}_{t-1}; \mu _\theta \left( {\textbf{x}}_t, t\right) , \Sigma _\theta \left( {\textbf{x}}_t, t\right) \right) . \end{aligned}$$

In order to train this model, let $p(x_0)$ learn the real data distribution $q(x_0)$, and also optimize the following variational upper bound according to the idea and theory of DDPM:

5 $$\begin{aligned} \begin{aligned} {\mathbb {E}}\left[ -\log p_\theta \left( {\textbf{x}}_0\right) \right]&\le {\mathbb {B}}_q\left[ -\log \frac{p_\theta \left( {\textbf{x}}_{0: T}\right) }{q\left( {\textbf{x}}_{1: T} \mid {\textbf{x}}_0\right) }\right] \\&={\mathbb {E}}_q\left[ -\log p\left( {\textbf{x}}_T\right) -\sum _{t \ge 1} \log \frac{p_\theta \left( {\textbf{x}}_{t-1} \mid {\textbf{x}}_t\right) }{q\left( {\textbf{x}}_t \mid {\textbf{x}}_{t-1}\right) }\right] \\&=-L_{\mathrm {VL.B}}. \end{aligned} \end{aligned}$$

Consistent with DDMP, this module chooses to let the network of the inverse process output random variables $\epsilon$, and use the predictive random variable method to train a model $\epsilon _\theta \left( x_t, t\right)$ to predict $\epsilon$, so the final loss function can be simplified as:

6 $$\begin{aligned} L_{\text {simple }}=E_{t, x_0, \epsilon }\left[ \left\| \epsilon -\epsilon _\theta \left( x_t, t\right) \right\| ^2\right] . \end{aligned}$$

Thus, through the diffusion forward process and reverse diffusion process, this model can obtain the addiction-related brain network through training, and learn the representation distribution of rat brain networks of different categories (normal saline, low concentration, and high concentration).

## Experiments

The fMRI data sets used in the experiment were divided into three groups, and the functional brain network was constructed from three different groups of rat fMRI scan data. After the original fMRI image data were preprocessed, the time series signals of the brain regions were extracted from the fMRI images according to the Wistar rat brain atlas. The functional connectivity matrix for each rat brain was obtained using Pearson correlation coefficients to calculate correlations between brain region time series. The following three evaluation groups were established: (1) high vs. saline, (2) low vs. saline, and (3) high vs. low.

Implementation detail: The PyTorch backend was used to implement the proposed GDRN. One Nvidia GeForce RTX 3090 was used to speed up the network’s training. The learning rate was set to 0.001, and the training epoch was set to 1000.

**Fig. 2:**
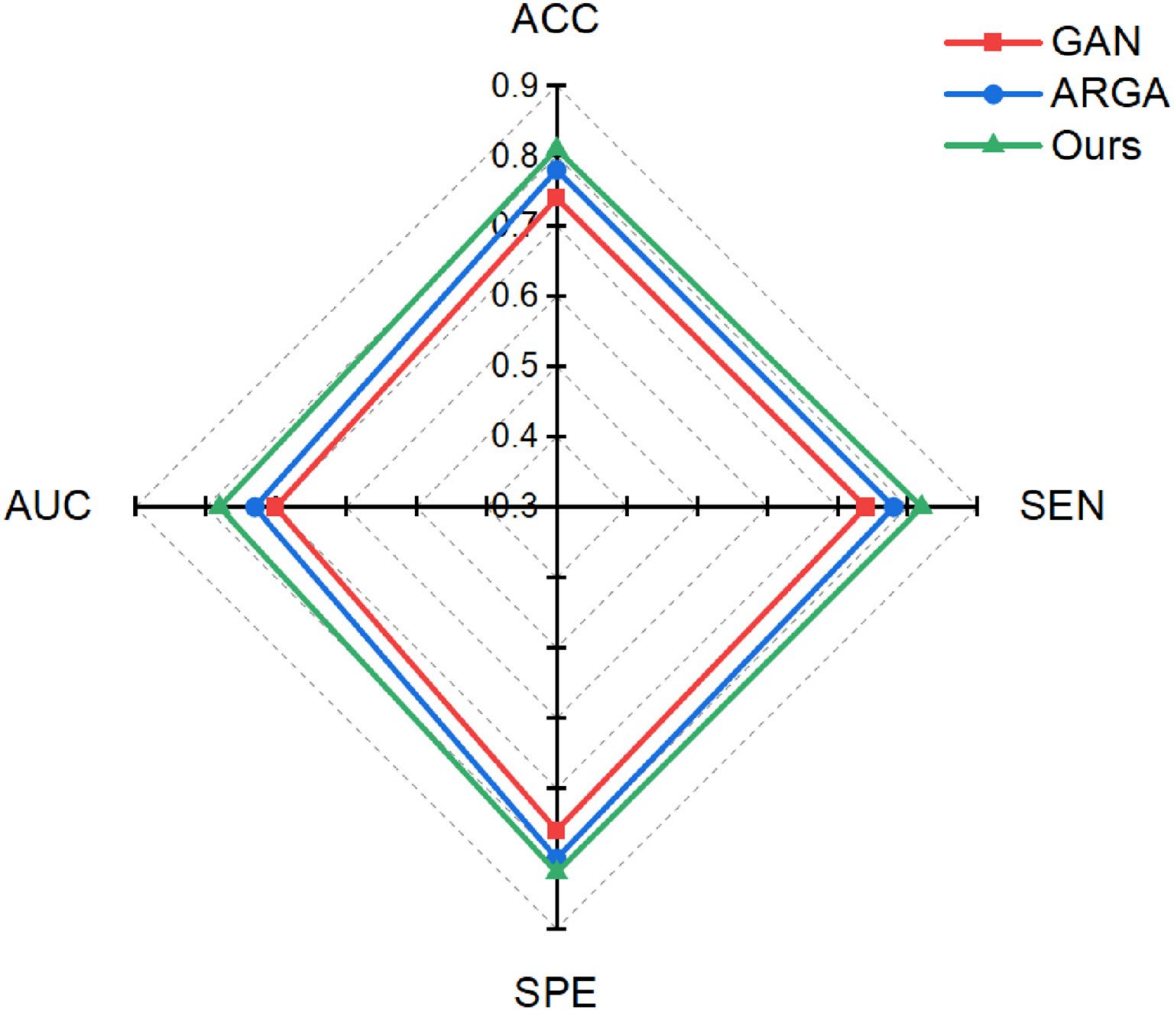
Performance of different models on datasets. Experimental results show that the proposed model outperforms other comparison methods

This study explores the brain networks that generate the resulting differences from nicotine injections. Inputting different types of rat brain network data, the model can use the learned information to generate more realistic network data. It was effective in alleviating the small-sample problem of addiction in rats. Classification experiments were carried out on the results, and the results are shown in Fig. 2. Experiments have verified that the model has good classification performance.

**Fig. 3:**
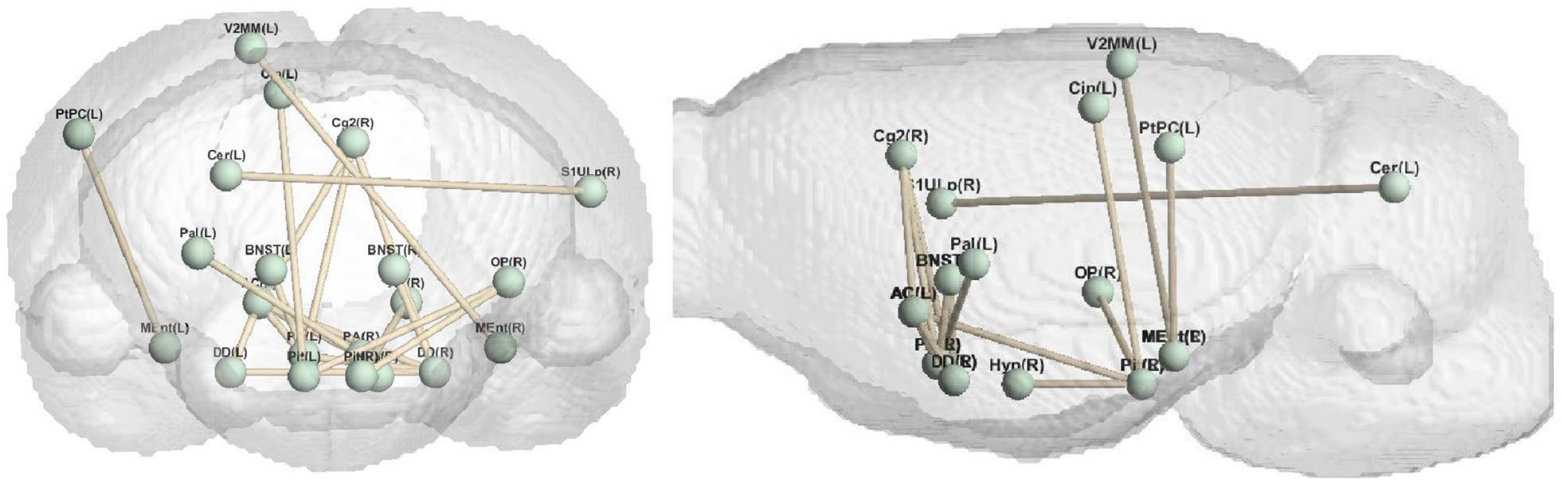
The brain connectivity with the most obvious differences between high concentration and normal saline

**Fig. 4:**
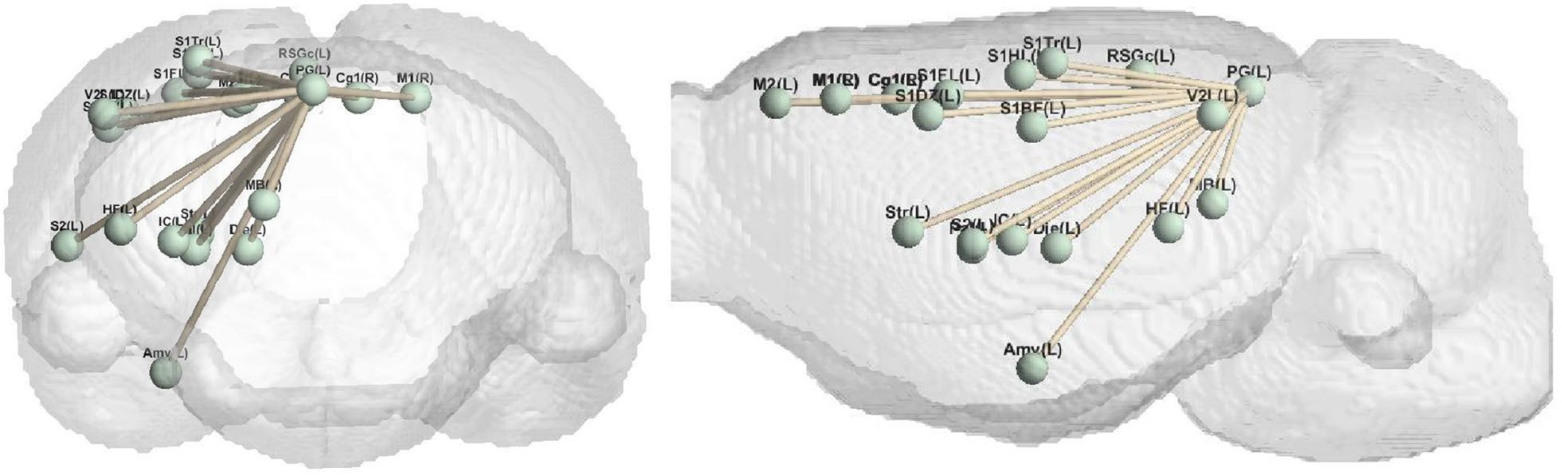
The brain connectivity with the most obvious differences between low concentration and normal saline

The model proposed in this paper can generate different types of brain networks, that is, reconstruct the functional connectivity matrix of the saline group, the low nicotine group, and the high nicotine group. The experimental results are analyzed from the perspective of brain connectivity. As depicted in Fig. 3, it illustrates the brain connections that exhibit the most pronounced differences between the high concentration group and the normal saline group across various experimental settings. These connections highlight the distinctive patterns of brain connectivity associated with high nicotine concentration exposure. Similarly, Fig. 4 displays the most prominent brain connections observed between the low-concentration nicotine group and the saline group, providing insights into the specific neural alterations resulting from low nicotine concentration exposure.

**Fig. 5:**
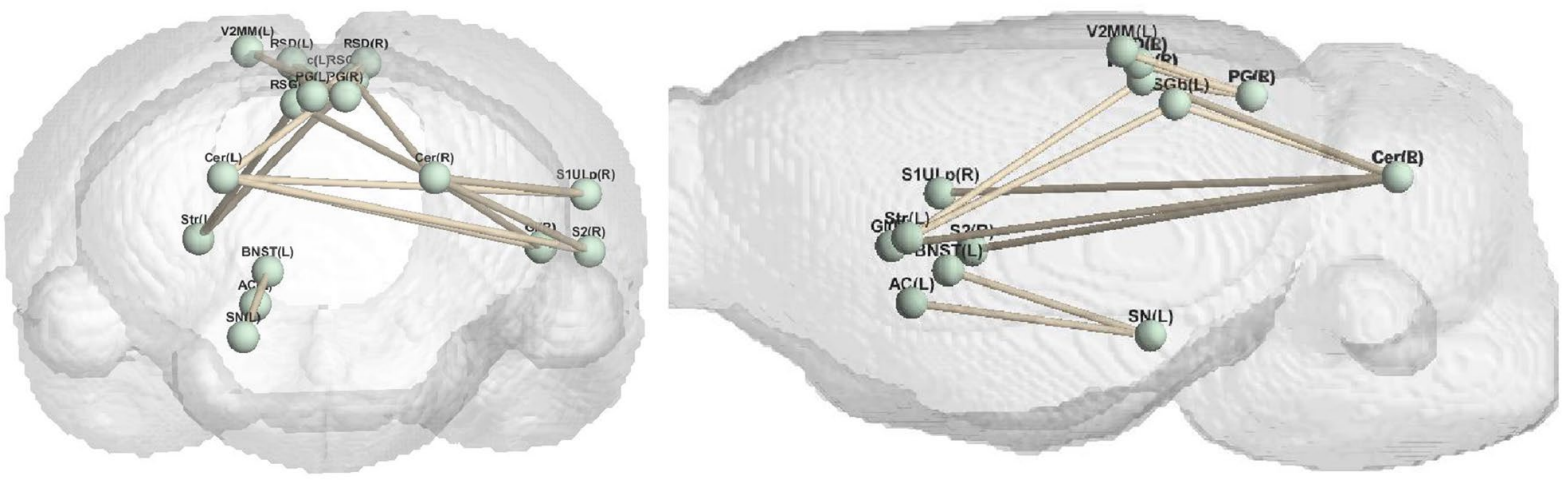
The brain connectivity with the most obvious differences between low concentration and high concentration

Additionally, we introduce Fig. 5, which presents a comparison of brain connections between the high and low nicotine concentration groups. This comparative analysis sheds light on the differential effects of high and low nicotine concentrations on brain connectivity, revealing distinct patterns of connectivity alterations associated with varying levels of nicotine exposure. The findings from Fig. 5 contribute to our understanding of the dose-dependent effects of nicotine addiction on the brain’s functional connectivity.

**Fig. 6:**
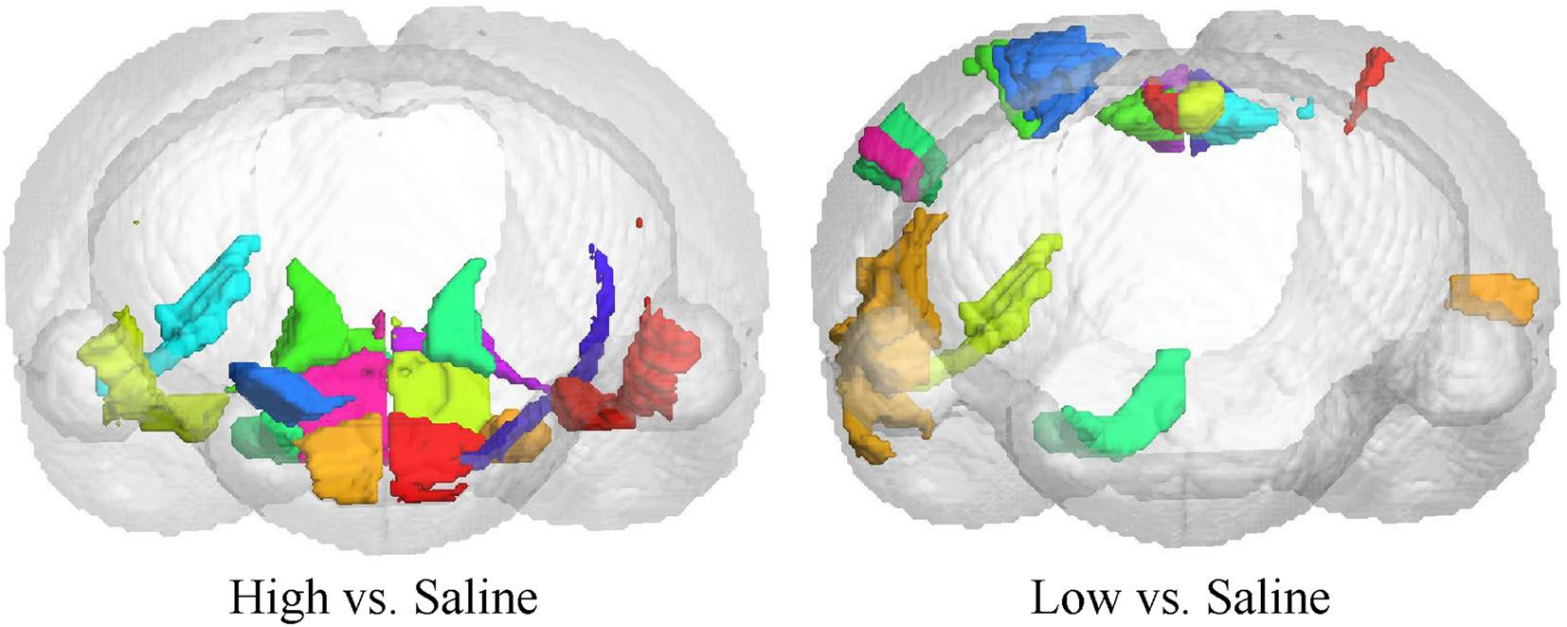
The brain regions with the most obvious differences between different groups

The brain regions with high weights, as identified in our analysis, exhibit strong associations with prior studies on addiction. These regions are visualized in Fig. 6, providing a comprehensive representation of their distribution. Notably, we observe a certain consistency between the distribution of these brain regions and the identified brain connections. In Fig. 6, it is evident that the low-concentration group tends to concentrate in the upper left part of the brain, while the high-concentration group shows a concentration in the middle and lower parts of the brain.

This spatial distribution suggests distinct patterns of brain involvement between the two groups, with specific brain regions being more prominently affected by different nicotine concentrations. Furthermore, the observed balance between the left and right hemispheres in terms of the distribution of these brain regions aligns with previous research findings. This consistency provides further support for the robustness of our results and reinforces the notion that nicotine addiction affects both hemispheres of the brain in a relatively balanced manner.

Tables 1, 2 and 3 show the top 15 brain regions with the most significant performance under different experimental settings. These tables provide valuable insights into the specific brain regions that are highly relevant in the context of addiction-related brain connectivity. Comparing the high-concentration nicotine group with the physiological saline control group, the most prominent brain regions associated with nicotine addiction in this comparison include pituitary_R, diagonal domain_R, medial entorhinal cortex_L, anterior commissure_L, diagonal domain_L, agranular insular cortex, ventral part_L, substantia nigra_L, optic pathways_R, anterior commissure_R, preoptic area_L, medial entorhinal cortex_R, pituitary_L, preoptic area_R, bed nucleus of the stria terminalis_L, bed nucleus of the stria terminalis_R.

**Table 1:** High vs. saline top 15 addiction-related brain regions

High vs. saline		
142	Pit_R [42]	Pituitary_R
110	DD_R [43]	Diagonal domain_R
64	MEnt_L	Medial entorhinal cortex_L
105	AC_L	Anterior commissure_L
111	DD_L [43]	Diagonal domain_L
48	AIV_L [44]	Agranular insular cortex, ventral part_L
103	SN_L [45]	Substantia nigra_L
146	OP_R	Optic pathways_R
104	AC_R	Anterior commissure_R
133	PA_L [46]	Preoptic area_L
18	MEnt_R	Medial entorhinal cortex_R
143	Pit_L [42]	Pituitary_L
132	PA_R [46]	Preoptic area_R
141	BNST_L [47]	Bed nucleus of the stria terminalis_L
140	BNST_R [47]	Bed nucleus of the stria terminalis_R

**Table 2:** Low vs. saline top 15 addiction-related brain regions

High vs. saline		
149	PG_L [48]	Pineal gland_L
14	GD_R [49]	Granular and dysgranular insular cortex_R
148	PG_R [48]	Pineal gland_R
71	RSGb_L	Retrosplenial granular cortex, b region_L
73	S1_L [50]	Primary somatosensory cortex_L
25	RSGb_R	Retrosplenial granular cortex, b region_R
87	V1M_L	Primary visual cortex, monocular area_L
26	RSGc_R	Retrosplenial granular cortex, c region_R
72	RSGc_L	Retrosplenial granular cortex, c region_L
67	PtPC_L [51]	Parietal cortex, posterior area, caudal part_L
34	S1Sh_R [50]	Primary somatosensory cortex, shoulder region_R
56	DLEnt_L [52]	Dorsolateral entorhinal cortex_L
48	AIV_L [44]	Agranular insular cortex, ventral part_L
80	S1Sh_L [50]	Primary somatosensory cortex, shoulder region_L
111	DD_L [43]	Diagonal domain_L

Comparing the low-concentration nicotine group with the physiological saline control group, the most prominent brain regions associated with nicotine addiction in this comparison include pineal gland_L, granular and dysgranular insular cortex_R, pineal gland_R, retrosplenial granular cortex, b region_L, primary somatosensory cortex_L, retrosplenial granular cortex, b region_R, primary visual cortex, monocular area_L, retrosplenial granular cortex, c region_R, retrosplenial granular cortex, c region_L, parietal cortex, posterior area, caudal part_L, primary somatosensory cortex, shoulder region_R, dorsolateral entorhinal cortex_L, agranular insular cortex, ventral part_L, primary somatosensory cortex, shoulder region_L, diagonal domain_L.

Comparing the low-concentration nicotine group with the high-concentration nicotine group, the most prominent brain regions associated with nicotine addiction in this comparison include retrosplenial granular cortex, c region_R, pineal gland_R, substantia nigra_L, pineal gland_L, ectorhinal cortex_L, anterior commissure_L, pituitary_R, retrosplenial granular cortex, b region_L, diagonal domain_R, cerebellum_R, cerebellum_L, bed nucleus of the stria terminalis_L, retrosplenial granular cortex, c region_L, retrosplenial dysgranular cortex_R, primary visual cortex, monocular area_R.

**Table 3:** High vs. low top 15 addiction-related brain regions

High vs. saline		
26	RSGc_R	Retrosplenial granular cortex, c region_R
148	PG_R [48]	Pineal gland_R
103	SN_L [45]	Substantia nigra_L
149	PG_L [48]	Pineal gland_L
57	Ect_L	Ectorhinal cortex_L
105	AC_L	Anterior commissure_L
142	Pit_R [42]	Pituitary_R
71	RSGb_L	Retrosplenial granular cortex, b region_L
110	DD_R [43]	Diagonal domain_R
136	Cer_R [53]	Cerebellum_R
137	Cer_L [53]	Cerebellum_L
141	BNST_L [47]	Bed nucleus of the stria terminalis_L
72	RSGc_L	Retrosplenial granular cortex, c region_L
24	RSD_R	Retrosplenial dysgranular cortex_R
41	V1M_R	Primary visual cortex, monocular area_R

**Fig. 7:**
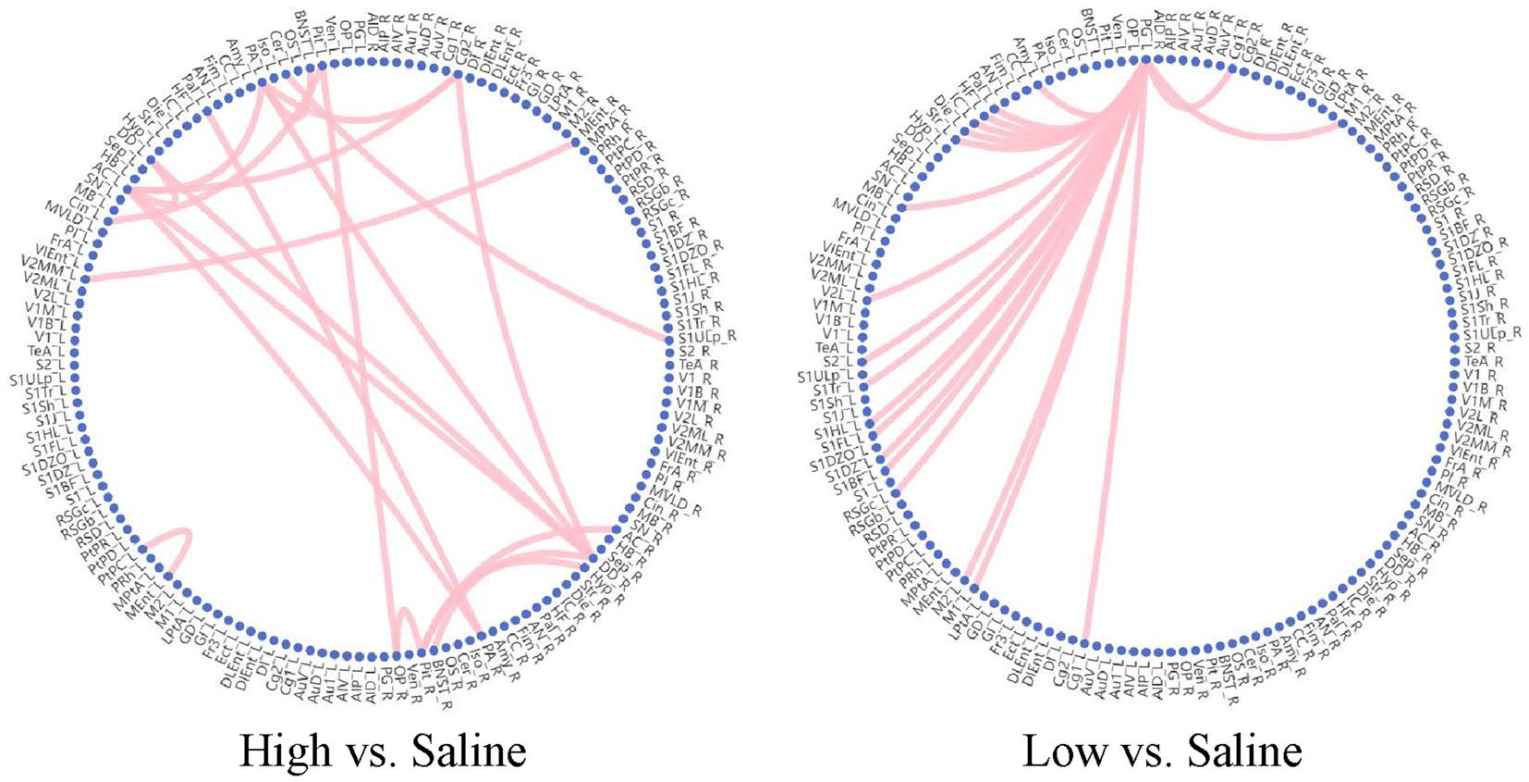
Nicotine addiction-related brain connections. The most pronounced brain connections at different nicotine concentrations. The threshold is set here

The observations from Fig. 7 reveal distinct patterns in the brain connections affected by low and high concentrations of nicotine. Specifically, the brain connections influenced by low-concentration nicotine are concentrated in the upper left region of the brain and exhibit a strong level of aggregation. In contrast, the brain connections affected by high concentrations of nicotine are concentrated in the lower right region of the brain and display a relatively uniform distribution.

This spatial pattern aligns with the underlying mechanisms of nicotine’s influence on the brain. When nicotine concentration is low, it tends to have a preferential impact on specific brain areas, resulting in a localized effect. As the concentration increases, the influence of nicotine spreads outward, eventually affecting a larger portion of the brain and leading to more widespread changes in brain connectivity.

The observed concentration of low-concentration nicotine effects in the upper left region of the brain suggests that specific brain areas may be particularly vulnerable or responsive to lower doses of nicotine. Conversely, the uniform distribution of high-concentration nicotine effects in the lower right region indicates a more widespread and generalized impact on brain connectivity. These findings provide valuable insights into the dose-dependent effects of nicotine on brain connectivity.

The study found that the brain regions and brain connections related to nicotine addiction at different concentrations have certain similarities, and these regions or connections can be confirmed in existing literature, which can prove the validity of the model. Moreover, compared with the normal saline group, there are certain differences in the most obvious brain connections between different concentrations. From another perspective, different addiction-related connections suggest that different doses may also correspond to different addiction mechanisms.

## Discussion

In the discussion section, we focus on the findings and implications of our proposed Graph Diffusion Reconstruction Network (GDRN) for capturing brain connectivity associated with nicotine addiction from fMRI data in rats. The GDRN incorporates a diffusion reconstruction module that effectively preserves the integrity of the data distribution in the latent space by reconstructing the training samples. This module enhances the model’s ability to reconstruct nicotine addiction-related brain networks, enabling the capture of subtle distribution differences and global correlations.

Part of the current work is related to our previous Brain Informatics work. The current research expands upon our previous work, encompassing a broader range of investigations. Additionally, we have incorporated experimental results to augment the depth and completeness of our analysis.

The most apparent brain connections in the high-concentration and saline groups were: (optic pathways_R, pituitary_R), [42] (diagonal domain_R, cingulate cortex, area 2_R), [43] (hypothalamus_R, pituitary_R), (parietal cortex, posterior area, caudal part_L, medial entorhinal cortex_L), [51] (pituitary_R, anterior commissure_R), (preoptic area_L, diagonal domain_R), [46] (anterior commissure_L, diagonal domain_L), (primary somatosensory cortex, upper lip region_R, cerebellum_L), [53] (anterior commissure_L, preoptic area_R), (secondary visual cortex, mediomedial area_L, medial entorhinal cortex_R), (cingulate cortex, area 2_R, anterior commissure_L), (preoptic area_L, cingulate cortex, area 2_R), (diagonal domain_L, diagonal domain_R), (diagonal domain_R, bed nucleus of the stria terminalis_R), [47] (diagonal domain_R, anterior commissure_L), (preoptic area_L, anterior commissure_L), (bed nucleus of the stria terminalis_L, preoptic area_L), (pituitary_L, cingulum_L), (pallidum_L, preoptic area_R), (pituitary_L, optic pathways_R).

The most apparent brain connections in the low concentration and normal saline groups were: (pineal gland_L, diencephalon_L), [48] (retrosplenial granular cortex, c region_L, pineal gland_L), (pineal gland_L, pallidum_L), (pineal gland_L, primary somatosensory cortex, hindlimb region_L), [50] (secondary visual cortex, lateral area_L, pineal gland_L), (hippocampal formation_L, pineal gland_L), (pineal gland_L, primary motor cortex_L), (pineal gland_L, primary somatosensory cortex, forelimb region_L), (pineal gland_L, primary somatosensory cortex, trunk region_L), (pineal gland_L, cingulate cortex, area 1_R), (primary somatosensory cortex, dysgranular zone_L, pineal gland_L), (secondary somatosensory cortex_L, pineal gland_L), (pineal gland_L, amygdala_L), (pineal gland_L, striatum_L), (primary somatosensory cortex, barrel field_L, pineal gland_L), (secondary motor cortex_L, pineal gland_L), (cingulate cortex, area 1_L, pineal gland_L), (pineal gland_L, internal capsule_L), (pineal gland_L, midbrain_L), (pineal gland_L, primary motor cortex_R).

The most apparent brain connections in the high concentration and low concentration groups were: (pineal gland_R, retrosplenial granular cortex, c region_L), [48] (primary somatosensory cortex, upper lip region_R, cerebellum_L), [50] (primary somatosensory cortex, upper lip region_R, cerebellum_R), [53] (cerebellum_L, secondary somatosensory cortex_R), (pineal gland_L, retrosplenial granular cortex, c region_L), (retrosplenial granular cortex, c region_R, cerebellum_R), (cerebellum_R, secondary somatosensory cortex_R), (cerebellum_L, granular insular cortex_R), [49] (anterior commissure_L, substantia nigra_L), [45] (cerebellum_L, retrosplenial granular cortex, c region_R), (cerebellum_R, granular insular cortex_R), (pineal gland_R, retrosplenial dysgranular cortex_L), (pineal gland_R, retrosplenial dysgranular cortex_R), (bed nucleus of the stria terminalis_L, substantia nigra_L), [47] (retrosplenial dysgranular cortex_R, pineal gland_L), (retrosplenial granular cortex, c region_R, striatum_L), (pineal gland_L, retrosplenial dysgranular cortex_L), (pineal gland_R, secondary visual cortex, mediomedial area_L), (retrosplenial granular cortex, b region_L, cerebellum_R), (retrosplenial granular cortex, b region_L, striatum_L).

**Fig. 8:**
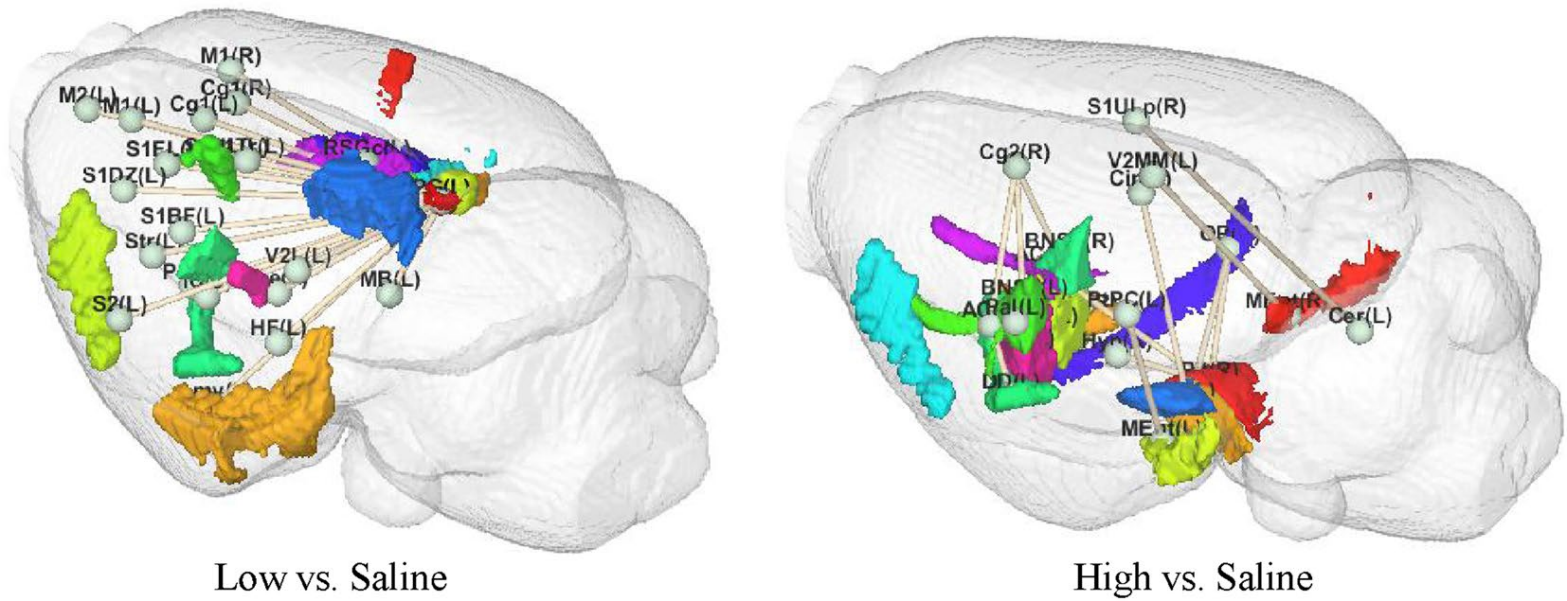
The consistency of addiction-related brain regions and brain connectivity from GDRN

In Fig. 8, a significant consistency can be observed between brain regions and brain connections. There is a close association between the activation of specific brain regions and the presence of corresponding brain connections. Notably, when certain brain regions display strong activation, the associated brain connections also exhibit noticeable enhancement. This consistency highlights the interconnectedness and interplay between brain regions and connections in forming complex brain functional networks.

Additionally, a spatial distribution consistency between the identified brain regions and connections is apparent. Specifically, related brain regions and connections tend to cluster in adjacent or proximate spatial locations. This spatial pattern suggests a locality feature in brain functional networks, where brain regions and connections with similar functions or interconnectedness are more likely to be in close physical proximity.

Our results demonstrate the remarkable performance of GDRN in generating nicotine-related connections. The majority of these results align with existing findings in neuroscience, providing validation for our approach. Additionally, we have identified novel nicotine-related brain connections and regions that have not been previously discovered, representing potential avenues for further exploration into the mechanisms underlying addiction.

By leveraging the power of fMRI data and the capabilities of GDRN, our study contributes to a deeper understanding of the complex brain connectivity patterns associated with nicotine addiction. The identified connections and regions provide valuable insights into the neural mechanisms involved in addiction processes and may serve as targets for future research and intervention strategies.

## Conclusion

This paper proposed a graph diffusion reconstruction network (GDRN) to capture brain connectivity associated with nicotine addiction from fMRI data in rats. The diffusion reconstruction module effectively maintains the unity of data distribution in the latent space through the reconstruction of training samples, and enhances the reconstruction ability of nicotine addiction-related brain networks. This module can make the model learn more subtle distribution differences and global correlations. This allows the model to effectively capture addiction-related brain connections. GDRN shows remarkable performance in nicotine-related connection generation. Most of the results obtained by the model are validated by existing work in neuroscience. The remaining results are considered as yet undiscovered nicotine-related brain connections and regions that can be used to explore mechanisms of addiction.

## Data Availability

The datasets analyzed during the current study are available from the corresponding author on reasonable request.
